# Mobile sonouroflowmetry using voiding sound and volume

**DOI:** 10.1038/s41598-021-90659-9

**Published:** 2021-05-27

**Authors:** Elie El Helou, Joy Naba, Karim Youssef, Georges Mjaess, Ghassan Sleilaty, Samar Helou

**Affiliations:** 1 Faculty of Medicine, Saint Joseph University, Beirut, Lebanon; 2 College of Engineering and Technology, American University of the Middle East, Egaila, Kuwait; 3 Global Center for Medical Engineering and Informatics, Osaka University, Osaka, Japan

**Keywords:** Urology, Bladder

## Abstract

Uroflowmetry (UF) is a common clinic-based non-invasive test to diagnose Lower Urinary Tract Dysfunction (LUTD). Accurate home-based uroflowmetry methods are needed to conveniently conduct repeated uroflowmetries when patients are physiologically ready to urinate. To this end, we propose and evaluate a novel mobile sonouroflowmetry (SUF) method that estimates the urinary flow rate from a sound signal recorded using a mobile phone. By linearly mapping the total sound energy to the total voided volume, the sound energy curve is transformed to a flow rate curve allowing the estimation of the flow rate over time. An evaluation using data from 44 healthy young men showed high similarity between the UF and SUF flow rates with a mixed-effects model correlation coefficient of 0.993 and a mean root mean square error of 2.37 ml/s. Maximum flow rates were estimated with an average absolute error of 2.41 ml/s. Future work on mobile uroflowmetry can use these results as an initial benchmark for flow rate estimation accuracy.

## Introduction

Lower Urinary Tract Dysfunction (LUTD) is a common complaint that can impact a patient’s quality of life and cause a substantial economic burden^1^. LUTD can be the result of numerous conditions including bladder outlet obstruction and over- or under-active bladder—which in turn can be caused by various conditions including benign prostatic hyperplasia and urethral stricture, neurologic diseases, diabetes, and pharmacologic treatment^2^. In recent decades, functional studies of the bladder and its outlet have become more widespread, rendering the assessment of LUTD more objective^3^. Among these diagnostic modalities, uroflowmetry (UF) is the easiest non-invasive LUTD assessment tool^4,5^, and is often considered sufficient for diagnosis and treatment of LUTD, along with history-taking and clinical examination^6^.

The important elements of a uroflowmetry test are the total voided volume (V_t_) (which should be > 150 ml), the maximum flow rate (Q_max_), and the curve of the flow rate (which should be bell shaped)^7^. Conducting a uroflowmetry involves the patient urinating in a flowmeter at a predetermined time. This process is unnatural and requires “on-demand” urination, often with low or very high bladder filling, instead of urinating when physiologically ready. This results in non-reproducible flow measurements^8,9^, hence the difficulty of diagnosis and therapeutic adaptation^10^. To get reproducible measurements of Q_max_, Q_max_ can be adjusted to V_t_ as a hyperbolic relationship exists between the two parameters^8^. However, the relationship between Q_max_ and V_t_ differs substantially between patients^8^. Therefore, to get accurate flow measurements, it has been recommended to repeat uroflowmetry tests for each patient, which requires multiple time-consuming and costly clinical visits^8,11–13^.

To address the limitations of conventional uroflowmetry, home-based uroflowmetry methods were proposed. To have reliable home measurements of Q_max_, it was estimated that as much as 25 flows are needed^14^. However, there are currently no cheap, practical, and accurate home-based methods that estimate the flow rate over time^15–17^. Promising approaches for home-based uroflowmetry are sound-based and vision-based^17^. A recent study showed that computer vision can be used to estimate the urinary flow rate. However, this requires the installation of a high-speed camera^18^. In comparison, mobile sonouroflowmetry (SUF) methods would be more practical, as they allow the extraction of urinary flow parameters from a mobile phone recording of the sound generated by the urine stream hitting a surface^10^. Even though it has long been recognized that the sound of a patient urinating can be used to diagnose LUTD^19–22^, sonouroflowmetry remains seldom studied in the literature.

Early experimental work validated the concept of mobile sonouroflowmetry by showing that the acoustic signatures of the sound generated by the urine stream are correlated with the urinary flow rate^23,24^; however, few studies have explored its feasibility and validity since 2011. Recent studies^10,25^ revalidated the basic concept and described high correlations between the intensity of the sound signal and the flow parameters measured using a uroflowmetry device. In 2021, Lee et al.^26^ evaluated the reliability of a smartphone-based sonouroflowmetry method in a population of male and female subjects with and without LUTD. The authors reported strong correlations between Q_max_, Q_avg_, and V_t_, and a strong visual correlation between flow curve patterns obtained with their SUF method and those obtained with a conventional UF device. However, the methods used to estimate the flow parameters were not fully described. In summary, existing results imply the existence of associations between UF and SUF flow parameters^10,26^ but do not detail the accuracy of the estimation methods. Moreover, the existing literature does not currently describe any reproducible SUF methods that allow the estimation of flow elements.

The aim of this study is to propose and evaluate the accuracy of a mobile sonouroflowmetry method. As inputs, the method takes the total voided volume (V_t_) and the mobile phone recording of the sound generated by the urine stream hitting water. The sound energy is assumed to be proportional to the flow rate at each moment, which implies that the total amount of sound energy is proportional to the total voided volume. Based on this assumption, the flow rate curve is generated and the Q_max_ is estimated. Finally, the accuracy of the sonouroflowmetry method is systematically examined and reported. In short, the contributions of this work are:

- A sonouroflowmetry method to estimate the flow rate from a sound signal recorded using a mobile phone.
- A systematic evaluation approach and an initial benchmark for mobile uroflowmetry methods.

## Methods

This study received approval from the institutional review board of Saint Joseph University, Beirut, Lebanon. The study was performed in accordance with the Declaration of Helsinki and written informed consent was obtained from all participants.

### Collection of real flow parameters and sound recordings

Volunteers were recruited for this study from Hotel Dieu de France, a large university hospital in Beirut, Lebanon, between December 2019 and November 2020. Those volunteers were: (1) male subjects aged between 18 and 65 years old; (2) with no lower urinary tract symptoms and no past history of urological or neurological comorbidities; (3) having autonomous voiding habits and (4) able to urinate in a standing position.

The recruited subjects underwent a standard uroflowmetry test using a uroflowmeter from Medtronic Medical Supplies (MMS), Dublin, Ireland. The uroflowmeter uses a high accuracy digital weight sensor and transmits the data to the computer via Bluetooth with a sampling frequency of 10 Hz. The uroflowmetry was combined with a synchronous sound recording using the native Voice Memos application on an Apple iPhone Xs Max (R), which was placed 100 cm away from the uroflowmetry machine. Each subject was asked to urinate, in a quiet room behind a curtain, inside a cup that was placed on the uroflowmeter and filled with 100 ml of tap water. The used cup was the original 1-l standardized plastic cup provided by the manufacturer–MMS—along with the uroflowmeter. The bottom diameter, top diameter, and height of the cup are 9.5, 12, and 16.5 cm, respectively. The subjects were asked to aim on the water inside the cup—not on its borders—while urinating, in order to produce a significant sound effect. The uroflowmeter, with the cup on top, were placed on the toilet lid to create a scenario that is easy to reproduce in home settings.

For each subject, the following variables were collected:

- Demographic parameters (age and height).
- Average and maximal urine flow rate (Q_avg_ and Q_max_), total voided volume (V_t_), voiding duration, and the flow rate curve from the uroflowmetry study.
- Mobile phone recording of the urination sound.

### Sound signal processing

#### Cropping and filtering

The recorded sounds of the urination were made with a sampling frequency ${f}_{s}$ of 48 kHz. The recording was initiated before the subject started urinating and was stopped after the subject finished. Therefore, the recordings were manually cropped to remove parts not corresponding to urination. When cropping the recordings, we took into consideration the variations in the sound signal, independently from the flowrate captured by the uroflowmeter. Each recording was used as an audio signal and processed as a sequence of samples. Background noise existed in the recordings; its low-frequency components were reduced using a third-order Butterworth high-pass filter with a cutoff frequency ${f}_{c}$ of 1.2 kHz. The obtained signals were used in the next processing stages.

#### Splitting into frames

Let *s* be one of the sound signals. *s* is decomposed into a sequence of consecutive time frames of equal durations. As each second corresponds to ${f}_{s}$ samples, a frame of duration ${f}_{d}$ seconds comprises of *nbs* = ${f}_{d}$ × ${f}_{s}$ samples and a recording with a duration of *d* seconds contains *nbf* = $\lfloor \frac{d}{{f}_{d}}\rfloor$ frames.

#### Calculating the sound energy

The measure E = ${\sum }_{i=1}^{nbs}\left|f(i)\right|$ is calculated for each frame, where *f(i)* is the value of the frame’s *i*th sample and |.| denotes the absolute value operator. Calculating E for all the frames of the signal *s* leads to *nbf* values of E reflecting the variations of the sound energy in the signal. Therefore, a signal of *nbf* frames will be represented by a sequence of *nbf* energy values, calculated according to the previous method.

### Estimation of flow parameters

**Figure 1:**
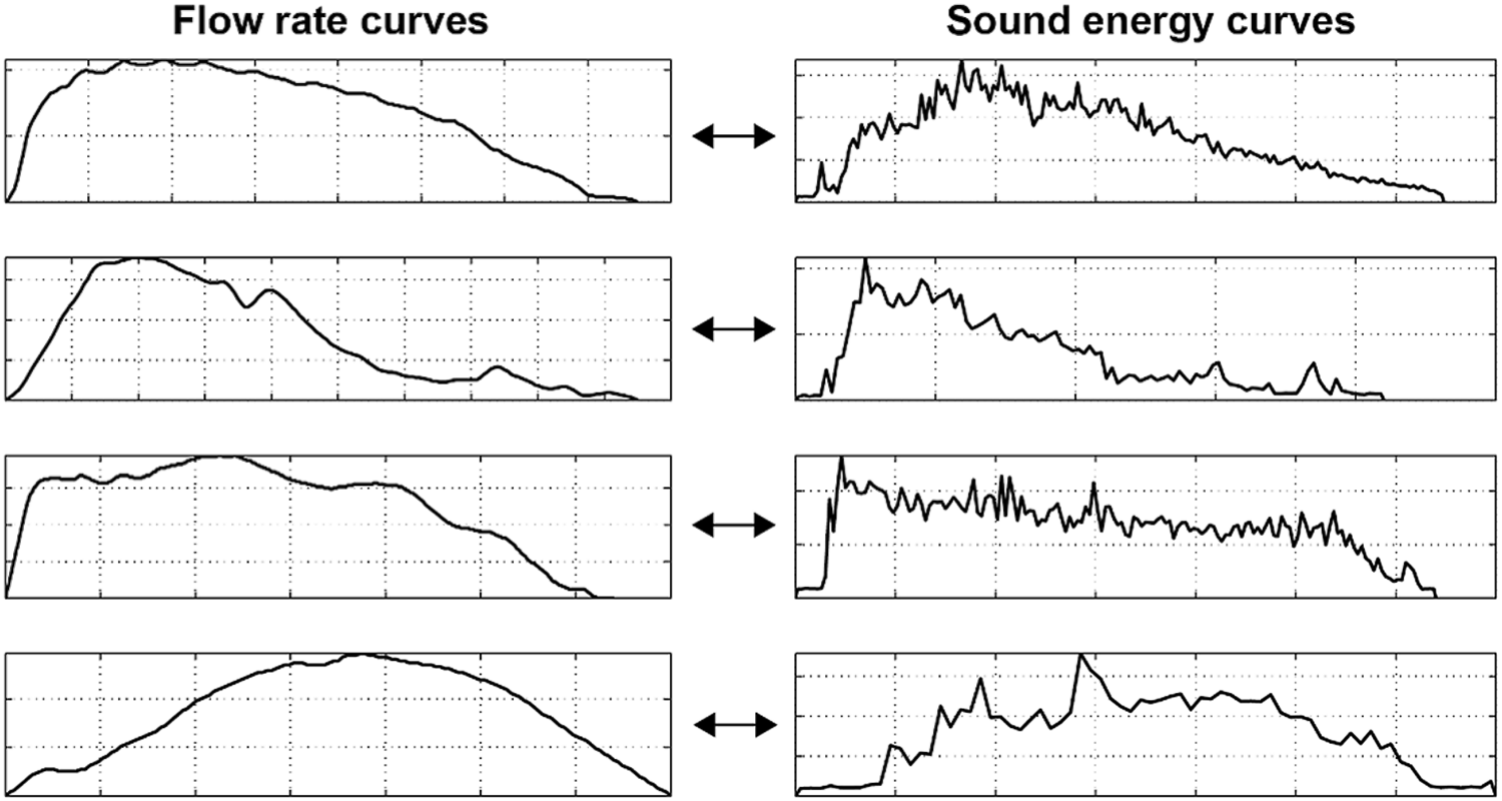
Flow rate and corresponding sound energy curves. The left-hand side graph shows the flow rate across time, and the right-hand side shows the corresponding sound energy across time. The y-scales in the graphs have different units and were scaled to visually highlight the similarity between the shapes of the curves.

Figure 1 shows four flow rate curves and their corresponding sound-based curves showing E in function of time. The left-hand side graph shows the flow rate curve, and the right-hand side shows the E curve. The y-scales in the graphs have different units and were scaled to visually highlight the similarity between the shapes of the curves. As seen in Fig. 1, E values change in conjunction with the flow rate. Therefore, we assume that the sound energy can be used for the estimation of the flow rate.

As observed, *E* varies in conjunction with the flow rate. Therefore, the area ${A}_{t}$ under the *E*-time curve (AUC_E_) varies in conjunction with the area under the flow rate-time curve (AUC_Flowrate_) which is equivalent to the total voided volume ${V}_{t}$. Normalizing the *E-*time curve by multiplying all the *E* values by the ratio $\frac{{V}_{t}}{{A}_{t}}$ rescales this curve and makes it a sound-based representation of the flow rate curve. Accordingly, the formula used to estimate the flow rate (*Q*) from the sound energy (*E*) is:

$$Q=E \times \frac{{V}_{t}}{{A}_{t}}.$$

Once the flow rate-time curve is obtained, Q_max_ can be calculated as the peak of the curve.

**Figure 2:**
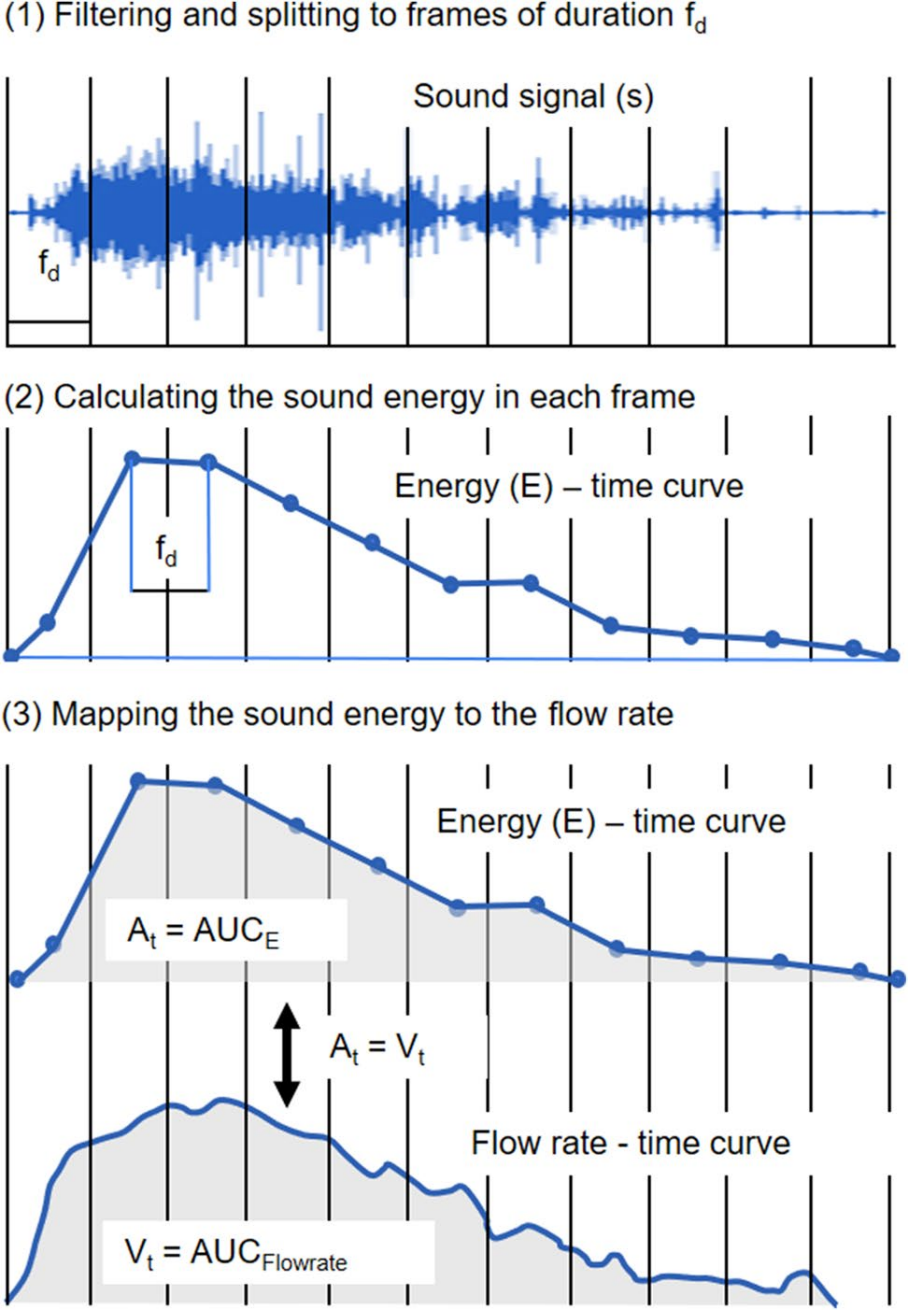
Transforming the sound signal to a flow rate-time curve. (1) The sound signal is split into frames of duration f_d_. (2) The energy is calculated for each frame and the energy-time curve is generated. This energy varies in conjunction with the flow rate and can be transformed into a flow rate curve. (3) The energy-time curve is linearly mapped to the flowrate-time curve by equating A_t_, the area under the energy curve (AUC_E_), to the total voided volume V_t_ which corresponds to the area under the flowrate curve (AUC_Flowrate_).

Figure 2 visually depicts the process of splitting the sound signal into frames, calculating the sound energy for each frame, and mapping the energy to the flow rate.

The area ${A}_{t}$ under the sound-based *E*-time curve is calculated as the sum of consecutive surfaces comprising it. These surfaces are triangular at both ends of the curve, and trapezoidal in between. The trapezoidal surfaces have the same width which is equal to the frame duration ${f}_{d}$ and heights equal to the corresponding consecutive *E* values. This can be seen in Fig. 2 (2) and (3) showing the sequence of *E* values as they make consecutive surface elements.

The calculated flow rates are highly dependent on the chosen frame duration. The effect of using different frame durations can be seen in Fig. S7 in the Online Appendix. Therefore, the optimal frame duration for estimating the overall flow rate curve and Q_max_ may differ. Accordingly, our experiments investigate the frame durations that result in high accuracy for the overall flow rate curve and Q_max_, separately.

## Results

### Collected dataset

Fifty-five subjects were recruited in the study. Recordings showing interfering sounds (speech, breathing, movements) were excluded. Finally, 44 subjects with a median age of 24 years old were included. Demographic and descriptive parameters of the population are presented in Table 1. Means and standard deviations (SD) were used for Q_max_, Q_avg_ and height (normal distribution based on Shapiro–Wilk and inspection of the Q–Q plots). Medians and interquartile range (IQR) were used for duration of voiding, voided volume, and age (non-normal distribution based on Shapiro–Wilk and inspection of the Q–Q plots).

**Table 1:** Descriptive and demographic parameters.

	Value	Minimum	Maximum
Age, years (median ± IQR)	24 ± 1.75	23	55
Height, cm (mean ± SD)	178.4 ± 7.0	163	191
Q_max_, ml/s (mean ± SD)	22.4 ± 7.7	7.5	43.5
Q_avg_, ml/s (mean ± SD)	12.4 ± 4.2	3.2	22.8
Voiding duration, s (median ± IQR)	37.0 ± 28.8	16	165
Voided volume, ml (median ± IQR)	365 ± 225.3	82	947

*SD* standard deviation, *IQR* inter-quartile range.

### Accuracy of flow rate estimation over time

The benchmarks for the performance of urodynamic equipment recommend a measurement bandwidth of 1 to 5 Hz, i.e., one measurement per 0.2 to 1 s for uroflowmetry devices^27^. Accordingly, we evaluate our method for five frame durations ranging from 0.2 to 1 s.

To study the correlation between the SUF and UF flow rate, we consider the flow rate curve to be a series of repeated flow measures and adopt a fixed-effect linear mixed-effects model. This approach allows us to account for the non-independence of the observations and for the unequal number of within-subject observations^28,29^. One no-intercept mixed model was developed for each frame duration, choosing an auto-regressive 1st order (AR1) covariance structure, and a restricted estimation maximum likelihood approach. Degrees of freedom estimation was approximated using the Kenward-Roger method. For each model, Schwarz’s Bayesian Information Criterion (BIC) was calculated. The results are reported in Table 2.

**Table 2:** Correlation between the SUF and UF flow rate.

Frame duration	Schwarz’s Bayesian criterion (BIC)	AR1 (rho)*
0.2 s	13,231.59	0.993
0.4 s	10,863.08	0.985
0.6 s	8665.11	0.976
0.8 s	7150.54	0.966
1 s	6118.56	0.957

*Calculated from the correlation matrix for estimates of covariance parameters.

The results show a high level of correlation between the SUF and UF flow rate values for the five frame durations. The correlation is almost perfect for a frame duration of 0.2 s (rho = 0.993), and slightly decreases with the increase of the frame duration. These results imply that the estimation of flow rate using the proposed SUF method is highly reliable. However, they do not imply that the UF and SUF flow rate curves have the same shape, i.e., are completely overlapping.

**Figure 3:**
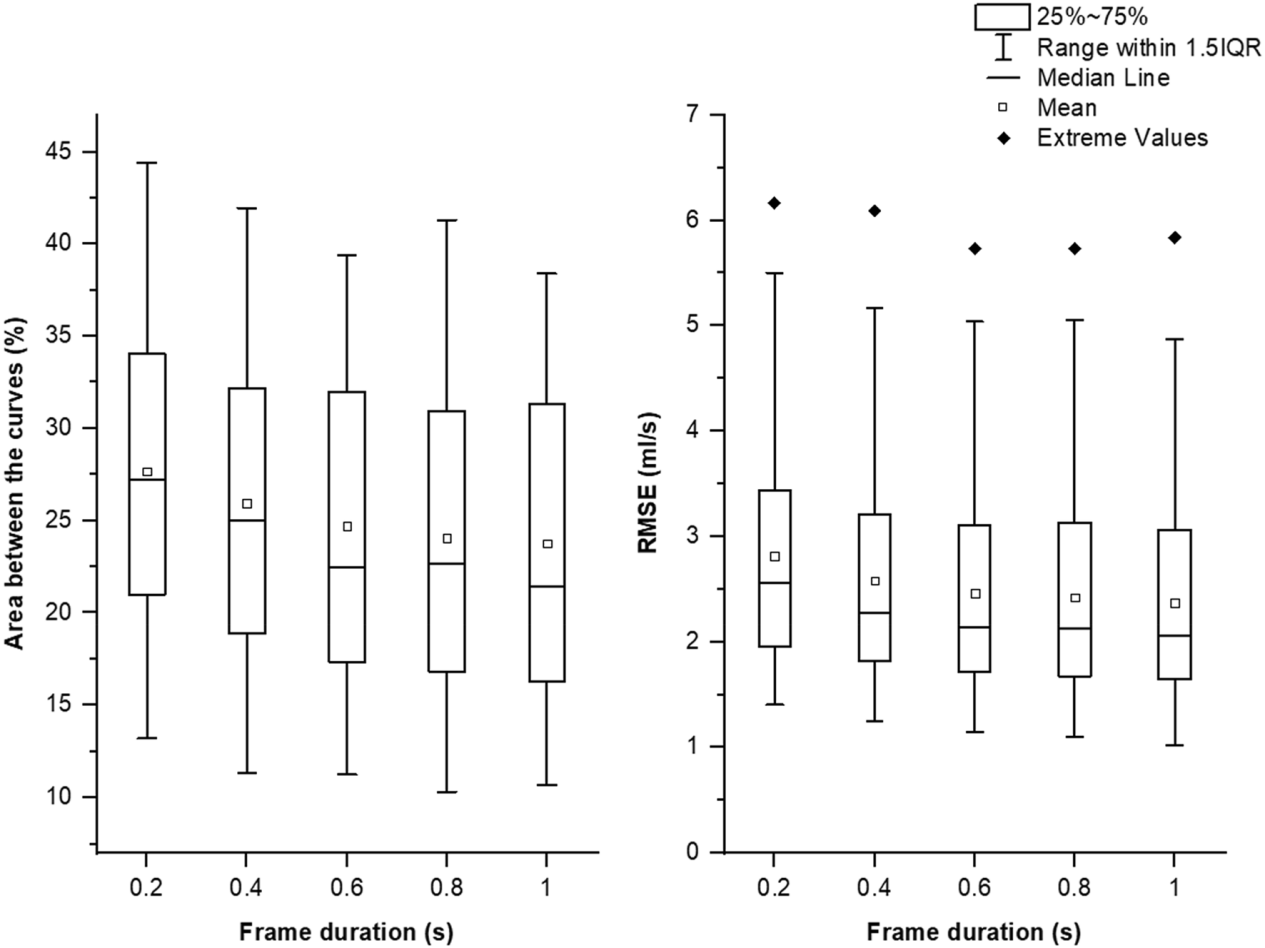
Comparison between the UF and SUF flow rate curves.

To assess the similarity between the shape of the UF and SUF flow rate curves, we report the area between the curves and the root mean square error (RMSE). The area between the curves was totalized and then normalized using the area under the UF flow rate curve. The results were generated by blinded processing as no pre-processing aiming to increase the alignment of the UF and SUF curves was conducted. Figure 3 shows the areas between the curves and the RMSEs and Table 3 shows their corresponding descriptive statistics.

**Table 3:** Comparison between the UF and SUF flow rate curves.

	Normalized area between the curves (%)				Root mean square error (RMSE) (ml/s)			
	Minimum	Maximum	Mean	SD	Minimum	Maximum	Mean	SD
0.2 s	13.21	44.40	27.67	8.12	1.40	6.17	2.81	1.13
0.4 s	11.36	41.97	25.95	8.10	1.25	6.09	2.58	1.09
0.6 s	11.26	39.39	24.69	7.92	1.15	5.73	2.46	1.06
0.8 s	10.31	41.29	24.08	7.97	1.10	5.73	2.42	1.08
1 s	10.70	38.40	23.76	8.04	1.02	5.84	2.37	1.08

*SD* standard deviation.

The results show that the similarity between the UF and SUF flow rate curves increases with the increase in frame durations. This may be due to the flattening of sound artifacts when a larger number of samples is considered in one frame. The mean RMSE and area between the curves decrease from 2.81 ml/s and 27.67% for a frame duration of 0.2 s to 2.37 ml/s and 23.76% for a frame duration of 1 s.

**Figure 4:**
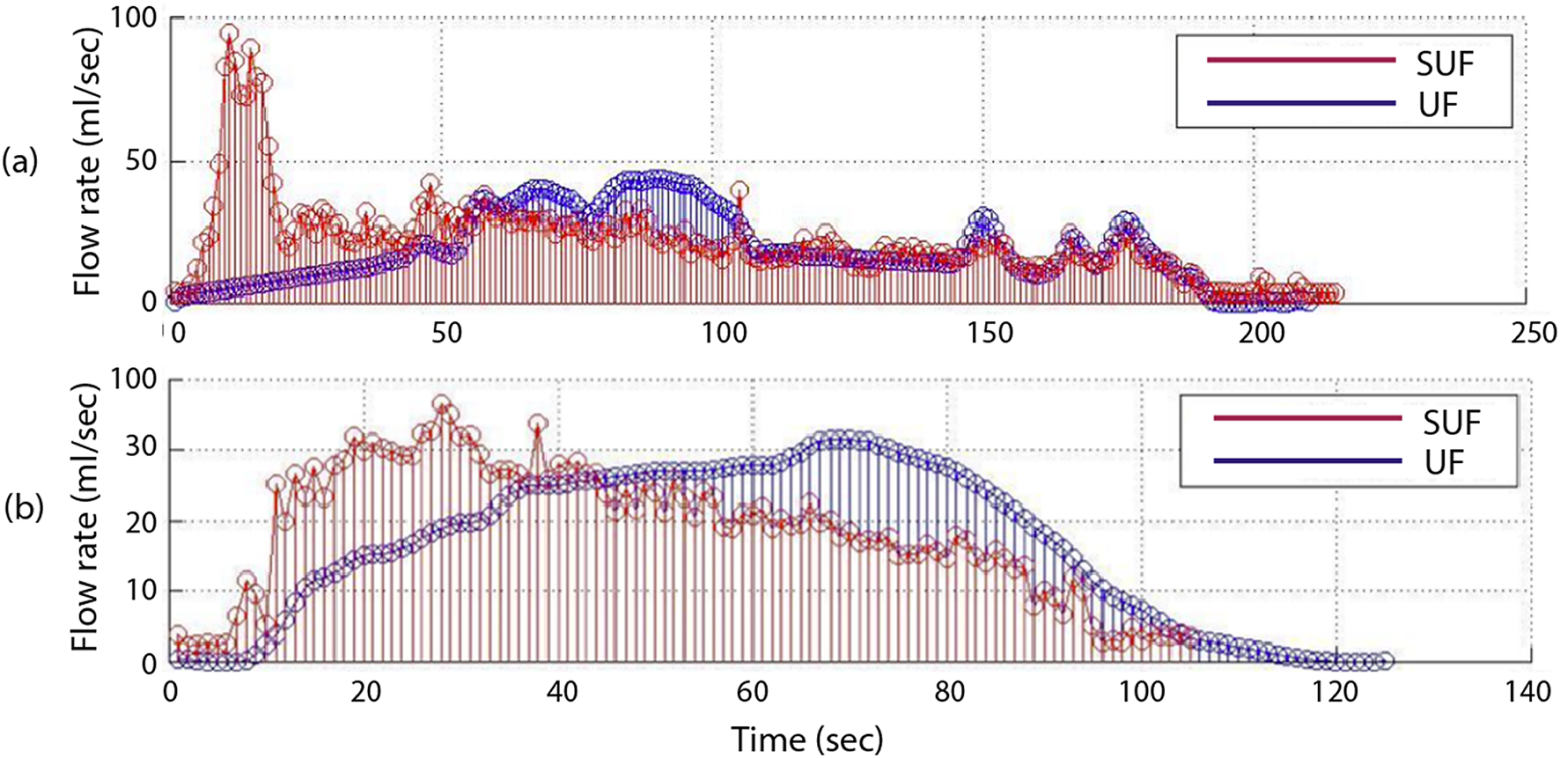
Cases with large non-overlaps between the UF and SUF flow rate curves. *UF* uroflowmetry, *SUF* sonouroflowmetry.

Two cases were found to be outliers with large non-overlaps between the UF and SUF curves. The two outlier cases are shown in Fig. 4 and offer insights into two main technical challenges that could be encountered with sonouroflowmetry.

The first issue is the potential presence of large sound artifacts as shown in Case (a). In Case (a), the SUF curve overshoots in the beginning possibly due to a manual urethral occlusion. Moreover, this subject voided a large volume causing the distance between the meatus and the water to majorly decrease during the voiding. This could have resulted in a relatively higher sound amplitude in the beginning of the recording. The second issue, shown in Case (b), is the overestimation of the flow rate at the beginning, which translates into an underestimation at a later time. This may be due to the low amount of water in the cup and its subsequent increase.

### Accuracy of Q_max_ estimation

**Figure 5:**
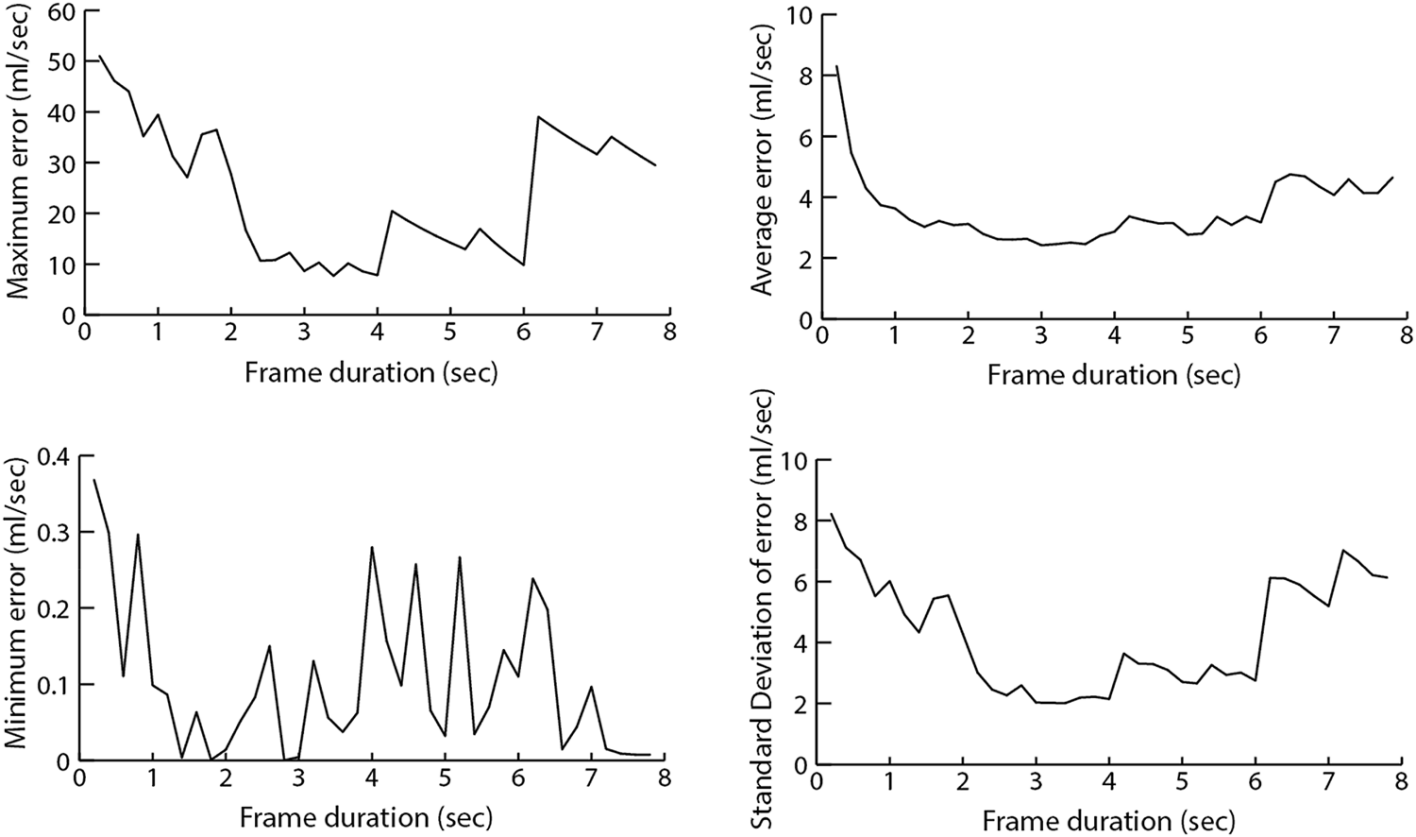
How Q_max_ error rate statistics vary with frame durations.

To assess the accuracy of the proposed method in estimating Q_max_, we report the errors associated with the estimations. Errors of estimation are calculated as the absolute values of the differences between UF and SUF based Q_max_. The average, standard deviation, minimum and maximum errors are calculated over the whole dataset. Figure 5 shows how error rates vary with frame duration ${f}_{d}$.

The results show that the error rates are high for small frame durations, decrease with the increase of frame duration, reach a minimum, and then increase again. Frame durations between 2.5 and 3.5 s provide the lowest maximum, minimum, and average error rates, in addition to the lowest amount of variation in error.

**Figure 6:**
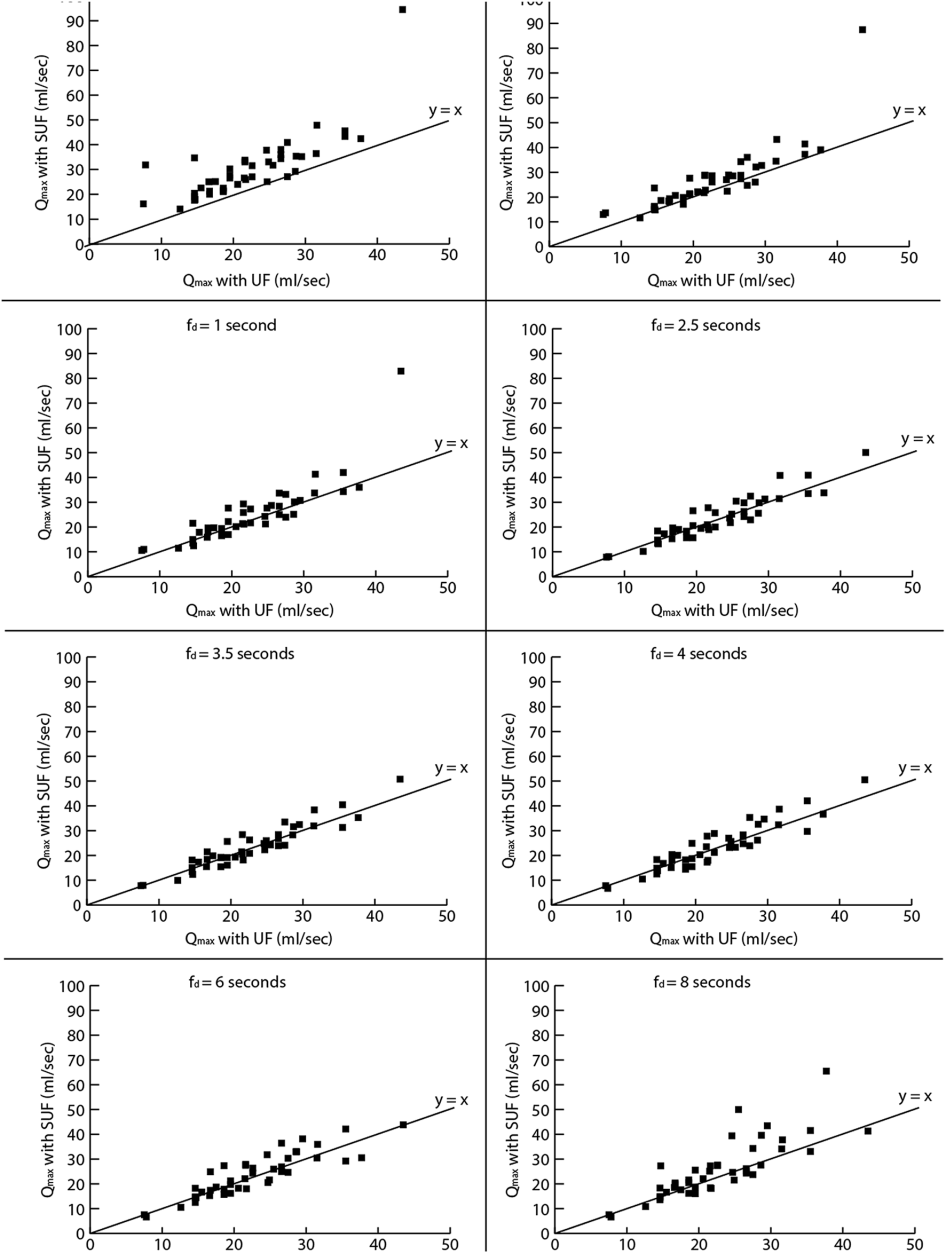
UF versus SUF Q_max_ for different frame durations.

Table 4 shows the statistics of error rates for select frame durations. Figure 6 shows UF versus SUF based Q_max_ measures for similar frame durations.

**Table 4:** Q_max_ error rate statistics for different frame durations.

Frame duration	Minimum error	Maximum error	Mean error	SD of error
0.2 s	0.37	51.02	8.30	8.22
0.4 s	0.30	46.15	5.45	7.11
0.6 s	0.11	44.03	4.29	6.71
1 s	0.10	39.42	3.63	6.01
2.5 s	0.06	9.26	2.50	2.12
3.5 s	0.08	7.23	2.41	2.01
4 s	0.28	7.83	2.87	2.15
6 s	0.11	9.84	3.17	2.75

*SD* Standard deviation.

## Discussion

This study proposed a reproducible mobile sonouroflowmetry method that allows the estimation of the flow rate over time. As inputs, the method requires the measure of the total voided volume and a mobile phone recording of the sound of the urine stream. By linearly mapping the total sound energy to the total voided volume, the sound energy curve was transformed to a flow rate curve, allowing the estimation of the flow rate over time.

The accuracy of the method was evaluated using data collected from 44 young healthy men. 80% (44 out of 55) of the collected measurements we considered in the evaluation due to the presence of interfering sounds in 20% (11 out of 55) of the recordings. The results showed a strong correlation and high similarity between the SUF and the UF flow rates with a mean RMSE of 2.37 ml/s for a frame duration of 1 s and a high accuracy in estimating Q_max_ with an average error of 2.41 ml/s for a frame duration of 3.5 s.

The average normalized area between the UF and SUF curves was over 20%. This could be attributed to (i) misalignments between the two curves caused by the manual cropping of the sound recordings and/or (ii) the rise of the water level inside the cup during the voiding which results in relatively higher energy levels in the beginning of the voiding. As the area under the curve is normalized using the total volume, an overestimation of flow rate in the beginning translates into an underestimation later on in time, which increases the area between the curves.

The study highlights the effect of the frame duration on the method’s performance. The use of shorter frames led to an overestimation of the Q_max_ and a higher RMSE. This is attributed to the impact of brisk sound artifacts on the energy estimation in small frames. The effect of these artifacts is diminished through the use of larger frames. However, when using large frames, we discarded the samples at the end of the sound signal that do not form a complete frame. This resulted in an overestimation of Q_max_ as the total volume was being fitted into a duration shorter than the real voiding duration. In our study, these two effects led to an overestimation of the Q_max_ in the majority of the recordings when using small and large frames. Future studies can examine ways to reduce the estimation errors attributed to sound artifacts and frame durations. A future direction to explore is the use of overlapping sliding frames which would allow the use of long frame durations while retaining the resolution of the flow rate curve. Sliding frames can also be useful for precisely detecting the start/end of voiding by minimizing the size of the segments to be discarded. In these cases, the optimal amount of overlap between the frames can be examined.

In addition to frame duration, the high-pass filter cutoff frequency f_c_ and the energy calculation method may affect the performance. The proposed method uses a cutoff frequency of 1.2 kHz and estimates the sound energy as the sum of the absolute values of the samples in a frame, rather than the sum of the squared values. These choices provided the best results following a series of trials, yet they may not be optimal. Further research is needed to inform the choice of cutoff frequencies and energy estimation methods for sonouroflowmetry applications.

Previous studies on sonouroflowmetry proved the feasibility of using mobile phone recordings to estimate flow parameters by reporting strong correlations between sound parameters and flow parameters^10,24^. However, only one previous study by Lee et al. aimed to estimate flow parameters by using a sound recording of a urine stream^26^. Therefore, we will mainly compare our study with the study of Lee et al.^26^. The method proposed in this study uses the sound recording of the urine stream in conjunction with the total voided volume in order to estimate the flow parameters. The awareness of the total voided volume was essential to our method as it allowed the transformation of the sound energy curve into a flow curve. Lee et al.’s method depended only on the sound recording of the urine stream without taking into consideration the total voided volume. However, the mapping of the sound features to flow parameters and the methods used to create the estimation models were not described. In other respects, the use of correlations and mean comparisons to assess the accuracy of the estimation models^26^ implies associations between truths and predictions, but does not provide an accuracy assessment of the models. As shown in our results, the accuracy of a sonouroflowmetry can be reported in terms of error rates that reflect the distance between real flow values and estimated flow values. Another evaluation aspect to consider is the similarity between flow curve patterns. As shown in our evaluation, the similarity between flow curves can be statistically assessed using a mixed-effects model approach and curve similarity measures. Visual comparisons are also necessary to assess whether the method’s results would be clinically interpreted by physicians similarly to the results of a traditional uroflowmetry. However, such comparison necessitates multiple experts and a randomized experiment using a heterogeneous dataset of healthy subjects and subjects with LUTD.

This study has some limitations. First, the total voided volume was not automatically estimated; the patient was required to measure it themselves while collecting their urine. We consider this limitation acceptable since it is a simple process and is already a common practice for patients filling a bladder diary. Moreover, portable measuring cups were shown to be practical and to provide accurate measures of voided volume^30^. Second, the proposed method does not automatically detect the start and the end of the flow. Therefore, Q_avg_ cannot be automatically estimated. In order to improve the usability of sonouroflowmetry methods, our future work will examine ways to automate the detection of the beginning and end of the flow and the estimation of voided volume. These methods will have to rely on features other than the sound energy such as spectral features because using sound energy does not allow us to distinguish between micturition and other activities that can produce sound such as talking, breathing, knocking objects, or undressing.

Another limitation of our study is the removal of the funnel normally used when conducting uroflowmetries with weight-based uroflowmeters. The funnel reduces the kinetic energy of the fluid and is needed to decrease the noise in the weight signal. Without the funnel, weight-based uroflowmeters will tend to overestimate the flow rate across time. This added error is difficult to assess because it depends on the velocity of the urine hitting the surface, which is related not only to the flow rate, but also to the diameter of the urethra. Since the removal of the funnel is necessary for SUF, this represents an inevitable limitation when comparing SUF with weight based UF.

In terms of applicability, our study did not examine the accuracy of SUF with low flow rates. However, some of the cases that were part of our experiment included low flow rates and intermittent flows. These flow rates and intermittencies were visible in the SUF curves as shown in Fig. S8 in the Online Appendix. Therefore, we assume that the proposed method would also perform well in those cases. In other respects, previous studies noted that different methods may be needed to estimate flow parameters for males and females^10,25,26^. Our study considered young men with no LUTD urinating in a standing position. Future work needs to consider women and people with LUTD urinating both in standing and sitting positions. Additionally, multiple factors may affect the amplitude of sound recordings such as the sound conditions in the room where the measure takes place, the type of mobile device used to perform the recording, the height of the patient, the size and shape of the toilet bowl, the amount of water inside, and the distance or position of the recording device, among others^22^. Future studies can examine the accuracy of sonouroflowmetry methods in relation to these various factors.

As for the implications of this work, uroflowmetry is currently restrained by two well-known limitations: (1) the high intra-subject variability of flow parameters^8,9^, and (2) the inability to reproduce the natural process of voiding, since the patient should urinate on demand. To address these two limitations, the only possible solution is to conduct multiple uroflowmetries for each patient, all when the patient is physiologically ready^8^. Home uroflowmetry methods would make this possible; however, there are currently no cheap, practical, and accurate home-based methods that estimate the flow rate over time^15,16^. In this case, a reliable mobile sonouroflowmetry application could fulfill this gap as it enables the patients to conduct multiple flow analyses, while at home, and for a relatively low cost. If applied to patients with LUTD, this would enable continuous home-monitoring while providing objective patient-generated data, a valuable addition to the commonly-requested frequency volume charts and bladder diaries^31,32^. Such application would also help promote telemedicine by limiting office visits, while providing objective parameters to the clinician, especially in critical situations such as the recent COVID-19 pandemic.

To conclude, we presented a new mobile sonouroflowmetry method that exploits the knowledge of the total voided volume to provide accurate estimations of the flow rate over time. The presented method estimates flow parameters from a sound recording of a urine stream hitting water using a frame-based energy calculation approach. Through a systematic evaluation approach, we showed that the method achieved a high level of accuracy in estimating the flow rate curve and the Q_max_. There are significant potential gains offered by mobile sonouroflowmetry, yet further work is needed to establish it as a replacement, or a complement, to clinic-based uroflowmetry. Future studies on sonouroflowmetry can use the results of this work as a benchmark and aim to provide reproducible improvements.

## Supplementary Information

Supplementary Information.

## Data Availability

The data that support the findings of this study are available from the authors upon reasonable request and with permission of Saint Joseph University, Beirut, Lebanon.
