# Supplementary material for: Mobile sonouroflowmetry using voiding sound and volume
Source: Sci Rep. 2021 May 27;11:11250. doi: 10.1038/s41598-021-90659-9 (PMC8159949; doi:10.1038/s41598-021-90659-9)
Supplement: Supplementary file 1 — Supplementary Information. [file 41598_2021_90659_MOESM1_ESM.docx]

**Appendix Material**


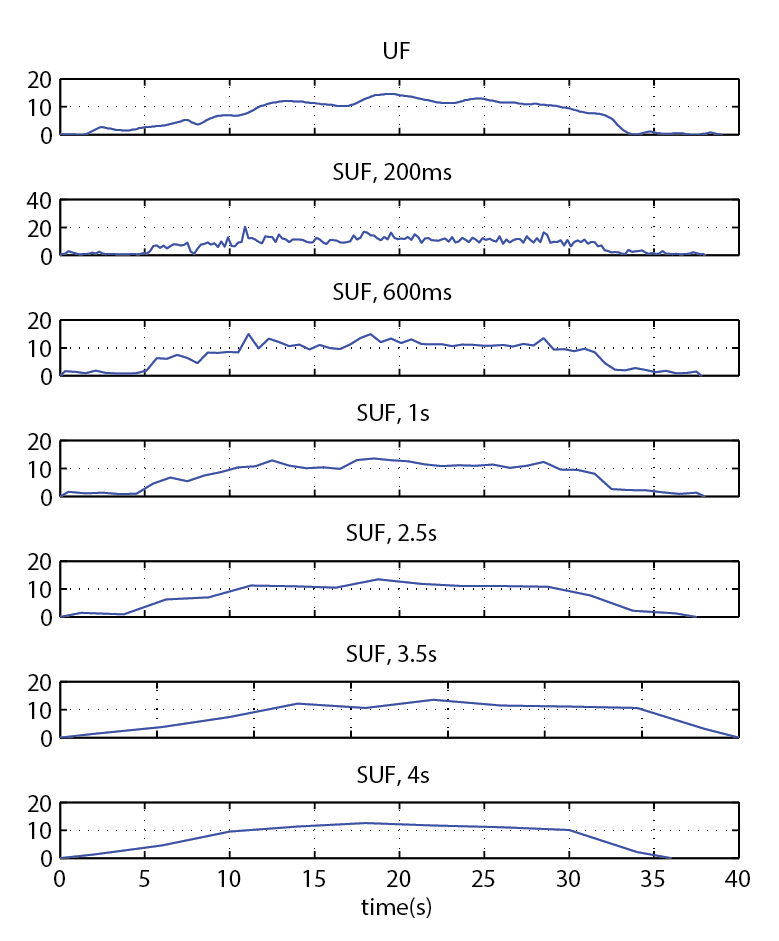


**Figure 7** Flow rate curves generated by the sonouroflowmetry (SUF) methods using different frame durations. The first graph shows the uroflowmetry (UF) based flow rate curve. The following graphs show the SUF based flow rate curves for frame durations of 200ms, 600ms, 1s, 2.5s, 3.5s, and 4s.


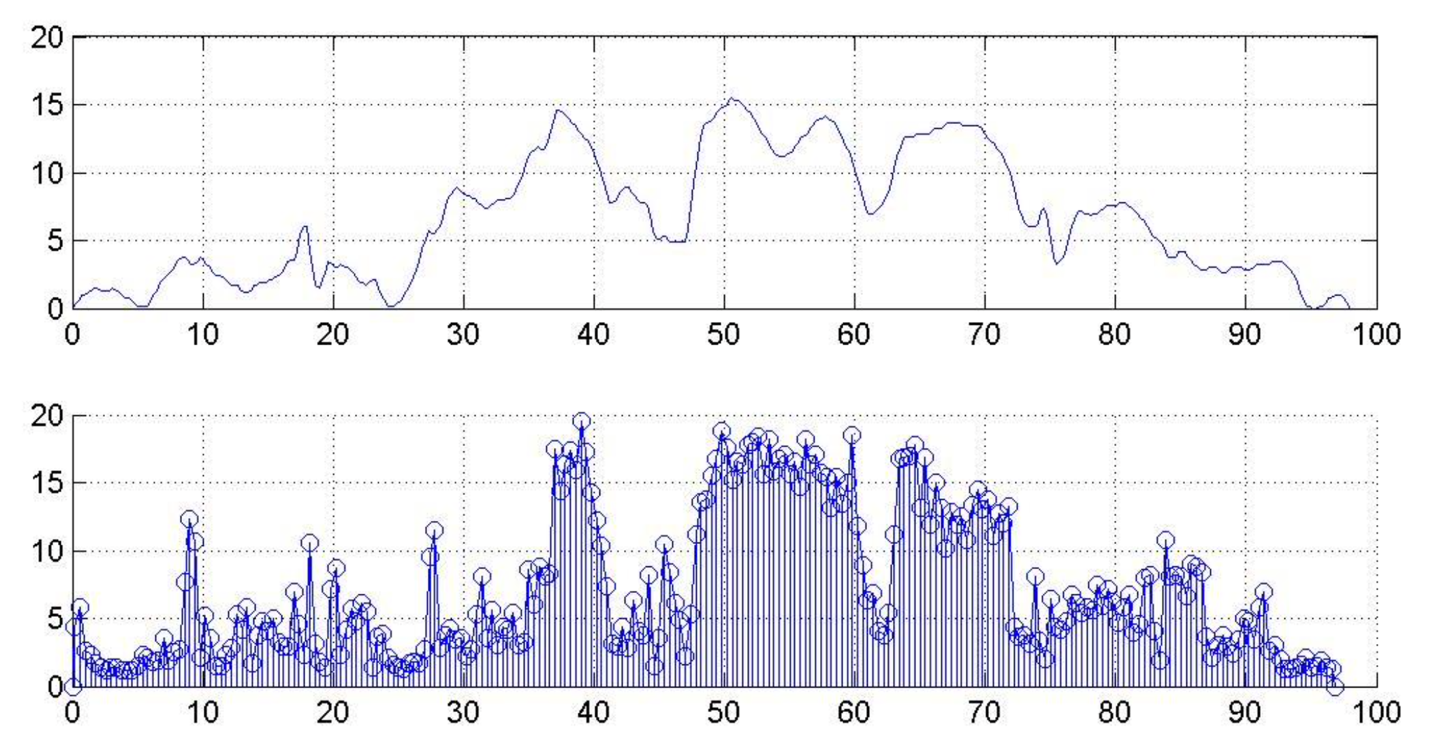
**Figure 8** A case with low flow rates and intermittent flows. The top graph shows the flow rate curve generated by conventional uroflowmetry (UF). The bottom graph shows the flow rate curve generated by the proposed sonouroflowmetry (SUF) with a frame duration f_d_ = 400 milliseconds.
